# Emergence of synchronized growth oscillations in filamentous fungi

**DOI:** 10.1098/rsif.2024.0574

**Published:** 2024-10-30

**Authors:** Praneet Prakash, Xue Jiang, Luke Richards, Zoe Schofield, Patrick Schäfer, Marco Polin, Orkun S. Soyer, Munehiro Asally

**Affiliations:** ^1^ School of Life Sciences, University of Warwick, Coventry, UK; ^2^ Department of Applied Mathematics and Theoretical Physics, University of Cambridge, Cambridge, UK; ^3^ School of Chemical Engineering, University of Birmingham, Birmingham, UK; ^4^ Department of Phytopathology, Justus Liebig University, Giessen, Germany; ^5^ Instituto Mediterráneo de Estudios Avanzados, IMEDEA UIB-CSIC, Esporles, Spain; ^6^ Department of Physics, University of Warwick, Coventry, UK

**Keywords:** emergence, synchronized growth, oscillation, filamentous fungi, soild microbe, growth dynamics

## Abstract

Many species of soil fungi grow in the form of branched networks that enable long-range communication and mass flow of nutrient. These networks play important roles in the soil ecosystem as a major decomposer of organic materials. While there have been investigations on the branching of the fungal networks, their long-term growth dynamics in space and time is still not very well understood. In this study, we monitor the spatio-temporal growth dynamics of the plant-promoting filamentous fungus *Serendipita indica* for several days in a controlled environment within a microfluidic chamber. We find that *S. indica* cells display synchronized growth oscillations with the onset of sporulation and at a period of 3 h. Quantifying this experimental synchronization of oscillatory dynamics, we show that the synchronization can be recapitulated by the nearest neighbour Kuramoto model with a millimetre-scale cell–cell coupling. The microfluidic set-up presented in this work may aid the future characterization of the molecular mechanisms of the cell–cell communication, which could lead to biophysical approaches for controlling fungi growth and reproductive sporulation in soil and plant health management.

## 1. Introduction

Filamentous fungi and some amoeboid have evolved a lifestyle where cells grow and spread in the form of a large-scale network [1]. Among these, mycelial fungi form centimetre-scale networks in the laboratory settings and can span as much as a few square kilometres in the wild [2,3]. These microbial networks can exhibit large-scale coordination over space and time. Filamentous fungi form branched growth patterns similar to *Physarum,* which is known to coordinate their movement over long distances [4], switch between behavioural states and are capable of encoding memory like neurons [5]. However, instead of the peristaltic spread characteristic of *Physarum*, the individual filaments of fungi are typically rigid and either grow over a surface or bore through the medium. They can regulate their growth and establish new connections or fuse branches to reorganize their network architecture [6–8].

Much of studies on fungal networks have focused on understanding the mechanisms behind the growth of individual fungal filaments, known as hyphae. The growth of hyphae is linked with internal hydrostatic pressure (turgor) changes, along with hyphae tip ‘softening’ and ‘hardening’ [9,10]. Individual hyphal tips are known to grow periodically, and synchronization of this periodic growth plays an important role in the fusion of hyphal filaments, and consequently the organization of network architecture [11]. Recently, hyphal growth studies in a microfluidic chip reported the presence of an autonomous clock in the hyphae of *Neurosopara crassa*, with synchronized growth oscillations having a period of approximately 20 h [12]. On the scale of hyphal networks, branching patterns and the dynamics of nutrient supply within the network have been explored [13–15]. These studies have revealed oscillatory spatial domains of nutrient acquisition within the network [7].

*Serendipita indica* (previously known as *Piriformospora indica*) is a fungal root endophyte. In addition to a saprophytic lifestyle on dead organic matter, *S. indica* forms symbiotic relationships with bacteria and plants [16,17]. Their relationship with plants encompasses nutrient exchange and protection of plants against biotic and abiotic environmental stress [18]. *Serendipita indica* can colonize a broad range of plants for its reproduction [19]. Despite their great ecological and agricultural significance, the growth dynamics of fungal endophytes are largely unexplored. It is unclear if their growth is determined locally or represents an orchestrated activity of the entire network.

In this work, we monitored the long-term growth of *S. indica* hyphal networks within a microfluidic device, from spore germination until the formation of new spores. The whole process lasted several days, during which we maintain a constant temperature and nutrient media supply in the device. Following the first few days of hyphal spreading, the culture exhibited an oscillatory growth pattern that gradually synchronized across the network. We recapitulate this synchronization using the Kuramoto model, a general model for synchronization of coupled oscillators [20–23]. The fitting of the model parameters to experimental observations on oscillation and synchronization dynamics suggests that the observed synchronization is best explained with the assumption of a cellular coupling within an area of approximately 1 mm^2^. These findings reveal a new phenomenon of synchronized growth dynamics in fungal hyphae with potential implications on nutrient cycling and fungal spore dispersion.

## 2. Material and methods

### 2.1. Spore harvesting

*Serendipita indica* was axenically cultivated on solid agar plates (1.5%) infused with glucose-based minimum essential growth media (MEM) at 30°C [16]. The MEM was composed of 0.2 mM MgSO_4_, 5 µM CaCl_2_, 10 µM FeSO_4_, 7.5 µM EDTA, 0.5 µM thiamine-HCl and 100 mM glucose in 1× MEM base media. Agar blocks from a 30-day-old fungal colony were plucked out and vigorously shaken to detach spores from hyphae filaments with 0.02% Tween20-deionized water in a falcon tube. The harvested spores were then filtered using nylon filters with a pore diameter of approximately 20 µm.

### 2.2. Loading *S. indica* spores in a microfluidic device

**Figure 1.**
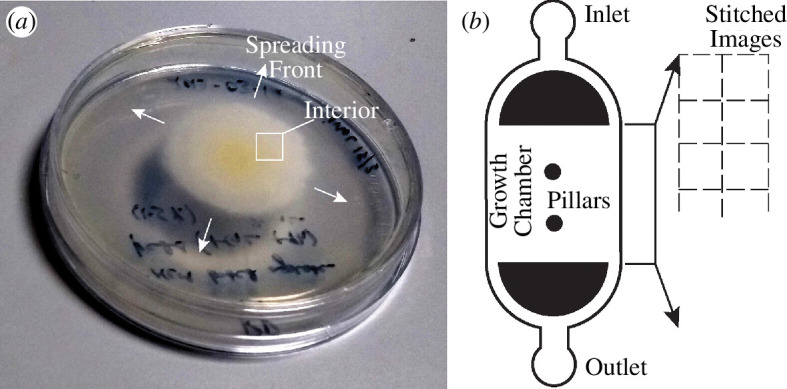
Growing filamentous fungi. (*a*) *Serendipita indica* colony cultivated on a 55 mm diameter Petri dish for one week. (*b*) Schematic of the microfluidic device with a growth chamber (H 6.5 mm × W 2.8 mm × D 14 µm) used for monitoring hyphal network growth. Two pillars in the centre prevent the collapse of the growth chamber. Microscopic images were recorded from 30 different locations covering the chamber, using a 4× microscope objective. Resulting image series from each location were stitched together to monitor chip-scale growth.

Fungal growth dynamics experiments were carried out in a custom-designed microfluidic device with a large central reservoir of area approximately 20 mm^2^ and a depth of 14 µm, i.e. nearly five times the diameter of a hyphal tip (figure 1*b*). Twin inlets and outlets maintain continuous supply of nutrient media and the two pillars in the centre prevent the reservoir from collapsing. To initiate the experiments, 2 ml of harvested spores (200 000 ml^−1^) were flushed into the microfluidic device maintained at 30°C. They were then supplied with minimum essential growth media at a flow rate of 0.1 µl min^−1^. A steady flow rate is maintained through the device by a syringe pump.

## 3. Time-lapse microscopy and the Kuramoto model

The spatio-temporal dynamics of the sample was monitored in brightfield using standard microscope objectives with 4× and 10× magnification. The open-source image processing software ImageJ, and custom-written Matlab codes were used to analyse the captured images [24]. Hyphal growth in the sample was indirectly measured by the decrease in image brightness since the microfluidic channel has a thin depth (14 µm). To analyse the synchronization of fungal growth dynamics, we estimated fresh growth by subtracting images at 90 min intervals. The differential brightfield intensity extracted through this methodology was subsequently subjected to detrending procedure, yielding a refined graphical representation of oscillation in spore growth rates. The Kuramoto model was simulated using Matlab and the order parameter was calculated as described in the method. See also the electronic supplementary material for more experimental details, detrending procedure and the source code use for analysis.

## 4. Results

### 4.1. Hyphal growth forms a network inside microfluidic devices

**Figure 2.**
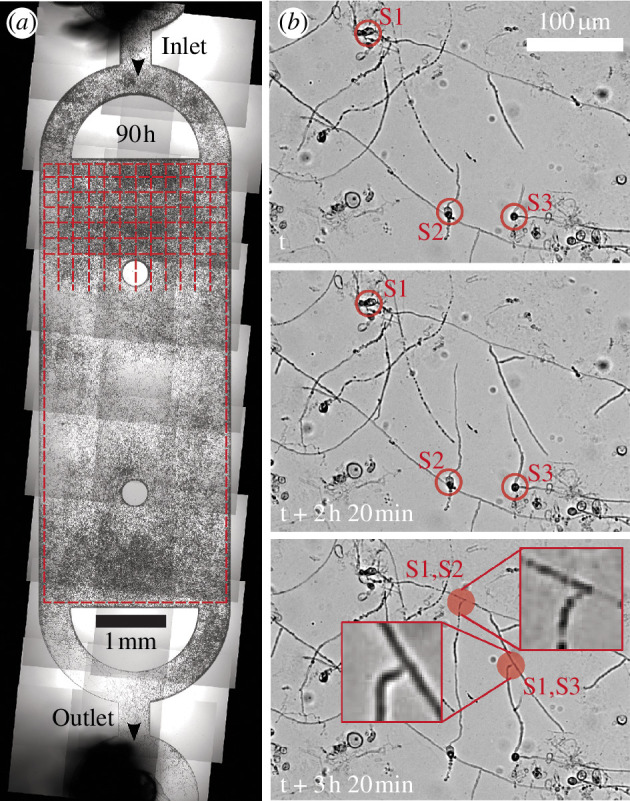
Stitched images of hyphal network in a microfluidic device. (*a*) Dynamics in the growth chamber is studied by dividing this area in 28 rows and 12 columns of equally sized squares. (*b*) Hyphae originating from various spores (S1, S2, S3) form a network. Bottom figure shows two growing hyphae terminating on nearby filament, possibly indicating hyphal filament fusion.

To investigate the spatio-temporal dynamics of fungal growth, *S. indica* spores were harvested from *S. indica* cultures grown on agar plates for one week (see §2 and figure 1*a*). Harvested spores were loaded in a custom-designed microfluidic device (figure 1*b*) and were supplied with growth media to enable their germination. We sequentially imaged the entire growth chamber to capture images at 20 spatial locations every 10 min for over 120 h. Images were then stitched together to obtain macroscale spatio-temporal pictures of hyphal networks (figure 2*a*). After inoculating the microfluidic chamber with *S. indica* spores, it takes between 6 and 24 h for spores to germinate, following which the hyphal network spreads rapidly (electronic supplementary material, Video 1). Figure 2*b* shows representative hyphal filaments from three distinct spores labelled S1, S2, S3. The diameter of hyphal tips was approximately 3 µm and they grew with an intermittent rate of approximately 0.3–0.4 µm min^−1^.

### 4.2. Fungal network displays growth oscillations

**Figure 3.**
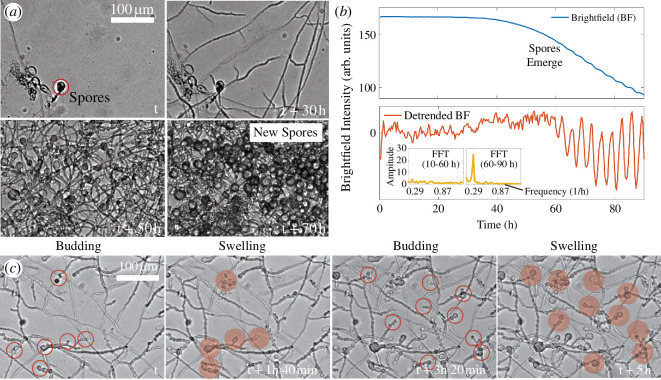
Periodic development of fungal spores. (*a*) Harvested spores germinating inside a microfluidic device. Over time hyphae grow and form a network followed by the formation of next-generation spores (timestamp, t+70 h). (*b*) Upper panel: 8-bit brightfield intensity of the entire frame as the fungal network grows in (*a*). Lower panel: detrended BF intensity displays the growth oscillations and corresponding fast Fourier transform (FFT) in the inset reveals the dominant frequency. (*c*) Next generation of spores periodically form (budding) leading to an increase in the collective growth of spores. Empty circles denote the budding of new spores, while filled circles indicate the swelling of spores. See the electronic supplementary material for a larger version of the images.

Following the expansion of hyphal network, new spores were formed typically after 48–72 h from the germination of the original spores (figure 3*a*). Within 12–14 h of first spores appearing, their diameter reached approximately 10 µm.

The brightfield intensity of the growing hyphal network shows a decline after approximately 40 h, indicating an increase in fungal biomass (figure 3*b*). Notably, after approximately 60 h, the decrease in brightfield intensity—signalling growth—is accompanied by clear periodic oscillations (figure 3*b*). To characterize the oscillation, we detrended the brightfield intensity by subtracting a moving average intensity calculated over a window of 6 h 40 min (figure 3*b*, lower panel). The fast Fourier transform (see electronic supplementary material for more details) of the detrended brightfield intensity showed a multi-modal spectrum until 60 h, as expected for signals that do not display clear oscillations, which eventually settled to a single frequency corresponding to an oscillation period of approximately 200 min (figure 3*b*, inset lower panel). This frequency appeared to be consistent with the periodic budding of spores, which also happened every approximately 200 min (see montage figure 3*c*).

### 4.3. Growth oscillations exhibit strong synchronization

**Figure 4.**
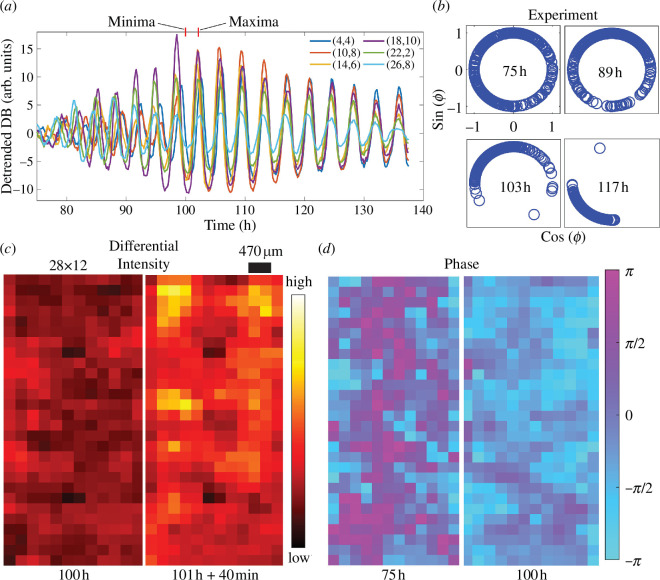
Synchronization of oscillations across a hyphal network. (*a*) Differential brightfield intensity at six spatial locations across the growth chamber. (*b*) Evolution of the experimental phase of oscillators starting from 75 h. (*c*) Heat map of differential brightfield intensity across different locations (28×12) within the microfluidic device each measuring 235×235 µm^2^ in size. (*d*) Phase of growth oscillations across the growth chamber near to the onset of oscillations (75 h) and after synchronization (100 h).

The dominance of a single frequency indicates that the oscillations synchronize across the hyphal networks. To characterize this synchronization process, we performed a ‘difference analysis’ as detailed in the electronic supplementary material, where we considered the changes in brightfield image intensity to estimate the fungal growth (electronic supplementary material, Video 2). To simplify the analysis of the spatio-temporal dynamics, the images were partitioned into 336 square regions (28×12) of size 235 × 235 µm. Each region, indicated by its position (i,j)(i=1,…,28;j=1,…,12), was then characterized by the differential intensity signal $\Delta I_{ij}\left(t\right)=I_{ij}\left(t+\Delta t\right)-I_{ij}(t)$ (figure 4). Following the temporal evolution of these signals reveals the onset of local oscillations at approximately 75 h after inoculation (figure 4*a*; average period of approximately 3.5 h). These quickly become synchronized across the whole device and remain synchronized until the end of observation period (approx. 140 h from inoculation). The amplitude of fungal growth rate is indicated by the intensity gradient of colours from yellow (high) to dark red (low) as shown in figure 4*c*. The two images display a heat map of differential brightfield intensity at minima and maxima after oscillations synchronize. Figure 4*d* shows the heat map of phases at 75 h, close to the onset of oscillations, and at 100 h when they are beginning to synchronize throughout the whole growth chamber (6.5 × 2.8 mm). To better characterize the synchronization, we estimated the instantaneous phase $\phi_{ij}(t)$ of the dominant frequency, derived from $\Delta I_{ij}\left(t\right)$ by Fourier transform. This phase characterizes the instantaneous state of each region. A full 2*π* cycle is then completed each time the system goes through equivalent points in the local detrended brightfield oscillation. Without loss of generality, we can take the peaks in the brightfield signal as references. The phase of the *n*th peak at position (i,j) can therefore be defined as $\phi_{ij}^{\left(n\right)}=2\pi n$ with a linear increase between successive peaks. Figure 4*b* shows that the distribution of local phases at 75 h close to the onset of oscillations, is spread across the whole set of possible values. However, as time passes the distribution becomes increasingly more compact, signifying a transition to global synchronization.

### 4.4. Large-scale synchronization is recapitulated by the short-range Kuramoto model

In order to analyse the synchronization dynamics, we now turn to Kuramoto model, a well-established minimal model for the synchronization of phase oscillators, with wide applicability in many biological systems and beyond [20]. Within the model, the phase of the oscillator at position (*i*, *j*) will evolve according to


(4.1) $$\frac{d\phi_{ij}}{dt}=\omega -\kappa \sum_{d_{ij}^{hk}}\sin\left(\phi_{ij}-\phi_{hk}\right),$$

where the intrinsic frequency ω=2πf is taken for simplicity to be independent of position, κ is the coupling constant between oscillators, and oscillators at (*i*, *j*) and (*h*, *k*) are coupled only if their distance $d_{ij}^{hk}$ is at most equal to the threshold separation *l*. We assumed all oscillators have the same natural frequency f=0.29 h^−1^, estimated as the average frequency observed after global synchronization at 102 h. Using the experimental phase values at 75 h as the initial phase, we simulated the time evolution of phase using the nearest neighbour Kuramoto model for threshold separation *l* given by equation (4.1).

**Figure 5.**
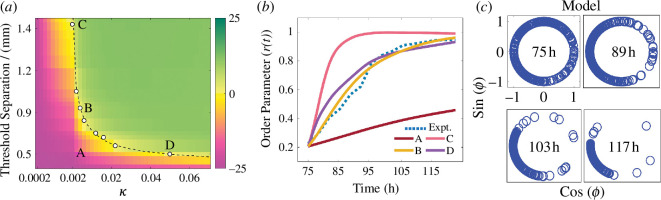
Kuramoto model recapitulates the fungal growth synchronization. (*a*) Heat map of the area difference between the order parameter obtained from experiments versus from simulations run with different parameters κ and l. Dashed line shows a theoretical relation between κ and l given by; $l=0.14\times \kappa^{-0.36}$, which shows a trendline for reasonable agreement with the order parameters obtained from experimental data. (*b*) Order parameter from experimental data (Expt.) and the Kuramoto model with the nearest neighbour coupling with four parameter sets (κ, l) in panel (*a*) are indicated as A–D. The location B, with a coupling coefficient κ=0.006 and threshold separation l=890 µm, closely retraces the experimental order parameter. (*c*) Evolution of the phase of oscillators from the model with κ=0.006 and threshold separation l=890 µm.

The phases can in turn be used to quantify this emergence of synchronization through the standard order parameter r(t), which measures the phase coherence defined as


(4.2) $$r\left(t\right)e^{i\psi \left(t\right)}=\frac{1}{N}\sum_{ji}e^{i\phi_{ij}\left(t\right)},$$

where$\;\phi_{ij}$ is the phase of oscillator at location (*i*, *j*), N is the number of locations, here 336. A comparison of evolved phases from the model and the experiments is made by computing the evolution of the order parameter over time using equation (4.2). We run the Kuramoto model simulations with the parameters 0.2 mm ≤l≤1.4mm and 0.0002≤κ≤0.07. To compare the simulation and experimental results, the integral area difference between the simulated order parameter and experimental curves was calculated for each of the simulation conditions (figure 5*a*, see electronic supplementary material). In this heat map, the pink region indicates limited synchronization, and the green region indicates stronger synchronization compared with the experimental data. The yellow region shows there is a good agreement between experimental and simulation-based evolution of order parameter curves. This parameter region can be approximated by a curve $l=0.14\kappa^{-0.36}$, describing the relation between the key parameters of coupling strength and spatial extent (figure 5*a*, dashed line). In other words, the experimental synchronization dynamics can be captured by the Kuramoto model, as long as the key parameters κ and l display the relation given by this equation. Figure 5*b* shows the time evolutions of the experimental order parameter and evolved phases at four locations (indicated as A–D in figure 5*a*). The location B (κ=0.006 and l=890 µm) most closely recapitulates the experimentally observed order parameter curve (figure 5*b*) suggesting that the global fungi growth synchronization across the microfluidic device of an area of 20 mm^2^ are best explained by a model assuming cell–cell coupling over approximately 1 mm. Keeping the same coupling coefficient as location B, a higher threshold separation (location C) overestimates the order parameter, while a lower threshold separation (location A) leads to underestimation. Likewise, maintaining the threshold separation as in location A and increasing the coupling coefficient to the value at location D yields a simulation closely matching the experimental order parameter.

The locations B and D are along the curve $l=0.14\kappa^{-0.36}$, which represents a generic exponential decay relationship between the threshold separation (l) for cell coupling and the corresponding coupling strength $\left(\kappa \right)$, recapitulating the synchronization of fungal growth. Furthermore, the theoretical evolution of the phase for location B (figure 5*c*) from the model aligns closely with the experimental phase evolution during the same time, as shown in figure 4*b*. Two independent experimental repeats showed a similar synchronization pattern, although there was a variation in the initial distribution of inoculated spores (electronic supplementary material, figure S4). Altogether these results show a robust synchronization of fungal growth which can be recapitulated by the Kuramoto model.

## 5. Discussion

We characterized the spatio-temporal growth dynamics of the plant beneficial filamentous fungus *S. indica* under controlled conditions. Using a custom microfluidic device, we maintained a constant nutrient supply and temperature conditions allowing us to image the growth of fungal colonies for nearly 6 days at a time. Fungi display growth under these conditions with the formation of a hyphal network, where an oscillatory growth dynamics emerges after 2.5 days of growth and with the onset of new spore formation. Intriguingly, these oscillations, initially synchronized only locally, eventually give rise to a global synchronization across a hyphal network covering an area of approximately 20 mm^2^.

The synchronization in several biological systems has been explained by physico-chemical coupling [21]. For example, collective thickness oscillation in plasmodium is driven by protoplasmic streaming [22], membrane potential oscillations in bacterial biofilm are regulated by spatially propagating waves of potassium [25], and gene expression oscillations in bacteria can be driven by quorum sensing molecules and synthetic transcription networks [26]. Emerging, synchronized oscillations have been previously reported in biofilms of the Gram-positive bacterium *Bacillus subtilis*, where nutrients are shared periodically between the inside and outside of the colony [27,28]. In that case, the oscillatory dynamics are shown to be mediated by electrical cell–cell signalling [29]. While the underlying biophysical and biochemical mechanism leading to presented case of synchronization in *S. indica* hyphal network is unknown, our analysis of the dynamics of synchronization against a Kuramoto model suggests the existence of cell–cell coupling at the scale of approximately 1 mm. Intriguingly, a recent study has demonstrated that Ca^2+^ signalling extends to the spatial distance of approximately 1.5 mm in *Aspergillus nidulans* mycelia network [30].

This study has a few technical limitations. While oscillation and synchronization were robustly observed in three experimental repeats, there were variations in initial spore number and distribution within the microfluidic devices, despite the use of same flow speed and same density of spores for loading (electronic supplementary material, figures S3 and S4). This is because the adhesion of spores was stochastic. In this study, fresh medium was constantly supplied to the microfluidic chamber to investigate the growth dynamics under a constant condition. However, there remains a possibility that biomass of fungi results in a complex flow pattern generating similarly complex chemical gradients. These could impact the network’s physiology. Better control over the initial spore distribution and visualization of nutrients in medium would be valuable for further investigations of biological mechanisms of oscillation and synchronization.

*Serendipita indica* colonize plants, form symbiotic relationships with bacteria and play an important part in the soil–plant ecosystem [31]. The oscillatory growth and resulting growth waves could transport nutrients from the leading front to the interior, which could facilitate a long-range exchange of nutrient resources. Such a property can be important in soil where materials are largely immobile. As fungi often inhabit constricted soil environments, collective growth could enable a stronger mechanical push, creating space for new spores. A future study could investigate the potential roles of the collective growth oscillations in the interspecies plant–fungi or bacteria–fungi interactions.

## Data Availability

Data are presented in the manuscript. The code is included in electronic supplementary file. Supplementary material is available online [32].
